# Down-regulated ciRS-7/up-regulated miR-7 axis aggravated cartilage degradation and autophagy defection by PI3K/AKT/mTOR activation mediated by IL-17A in osteoarthritis

**DOI:** 10.18632/aging.103731

**Published:** 2020-10-25

**Authors:** Xindie Zhou, Jin Li, Yuanshuai Zhou, Zhicheng Yang, Haoyu Yang, Dong Li, Junjie Zhang, Yi Zhang, Nanwei Xu, Yong Huang, Lifeng Jiang

**Affiliations:** 1 Department of Orthopedics, The Affiliated Changzhou No.2 People’s Hospital of Nanjing Medical University, Changzhou 213000, China; 2 Department of Orthopedic Surgery, The Second Affiliated Hospital of Jiaxing University, Jiaxing 314000, China; 3 Suzhou Institute of Biomedical Engineering and Technology, Chinese Academy of Sciences, Suzhou 215000, China; 4 Department of Orthopedic Surgery, The Second Affiliated Hospital, Zhejiang University School of Medicine, Hangzhou 310000, China

**Keywords:** ciRS-7, miR-7, autophagy, osteoarthritis, IL-17A

## Abstract

Osteoarthritis (OA) is one of the most painful and widespread chronic degenerative joint diseases and is characterized by destructed articular cartilage and inflamed joints. Previously, our findings indicated that circular RNA ciRS-7 (ciRS-7)/microRNA 7 (miR-7) axis is abnormally expressed in OA, and regulates proliferation, inflammatory responses, and apoptosis of interleukin-1β (IL-1β)-stimulated chondrocytes. However, its underlying role in OA remains unknown.

In this study, we first validated cartilage degradation and defection of autophagy in samples of OA patients. IL-1β initially stimulated autophagy of chondrocytes, and ultimately significantly suppressed autophagy. Upregulated ciRS-7/down-regulated miR-7 aggravated IL-1β-induced cartilage degradation, and restrained autophagy *in vitro*. Gene sequencing and bioinformatics analysis performed on a control group, IL-1β group, and IL-1β+miR-7-mimics group demonstrated that seven of the most significant mRNA candidates were enriched in the interleukin-17 (IL-17) signaling pathway. Increased IL-17A levels were also observed by qRT-PCR and ELISA. In addition, it was revealed that the ciRS-7/miR-7 axis ameliorated cartilage degradation and defection of autophagy by PI3K/AKT/mTOR activation in IL-1β-induced chondrocytes. Furthermore, an OA model was established in rats with medial meniscus destabilization. miR-7-siRNA-expressing lentiviruses alleviated surgical resection-induced cartilage destruction of OA mice, whereas miR-7 mimics worsened the effects. Thus, these findings revealed that the mechanism of the ciRS-7/miR-7 axis involved regulating OA progression and provided valuable directions for OA treatment.

## INTRODUCTION

Osteoarthritis (OA) is one of the most painful and widespread chronic degenerative joint diseases and is characterized by progressive articular cartilage degradation, synovial inflammation, and subchondral bone damage, which ultimately leads to physical disability and a decline in the quality of life [1, 2]. At present, effective treatment of OA is limited to pain management, including non-steroidal anti-inflammatory drugs (NSAIDs), cyclooxygenase 2 (COX-2) inhibitors, steroids, hyaluronic acid, etc., however, these treatments do not reverse the loss of articular cartilage [3]. In the terminal phase of OA, total joint replacement (TJA) is often required [4]. In many previous studies, the causes of OA revealed joint damage, heredity, obesity, aging, and inflammation, however, the pathogenesis of OA is complex and not fully understood [5, 6]. Chondrocytes are the only cell type in cartilage that is necessary for cartilage homeostasis and extracellular matrix integrity [7]. In several reports, it has been shown that genetic and epigenetic changes in chondrocytes are key factors of OA pathogenesis. Therefore, it is important to delve into the pathogenesis of OA, which helps to find novel therapeutic targets and methods for treating the disease [8, 9].

One factor that may be associated with the molecular mechanisms underlying OA is the regulation by microRNAs (miRNAs) [10, 11]. MiRNAs are a class of naturally occurring small non-coding RNAs of roughly 20-22 nucleotides that are present in eukaryotes, and regulate post-transcriptional inheritance by interacting with the 3'-untranslated region (UTR). Unlike other signaling regulators, each miRNA may control a large number of functionally related genes [12]. Increasing evidence has indicated that miRNAs are often dysregulated in human inflammatory diseases (including OA), and may play different roles [13]. Several miRNAs have been identified that are abnormally expressed in OA, including miR-9 and miR-140 [14–16]. Therefore, studying aberrant miRNAs in OA are important for exploring the underlying molecular mechanisms of OA. We have previously shown that expression of the ciRS-7/miR-7 axis in OA is abnormal, and has different regulatory effects on proliferation, inflammatory responses, and apoptosis of IL-1β-stimulated chondrocytes [17]. However, the underlying mechanism of action is not clear.

Autophagy is a catabolic process that degrades cellular components via a lysosomal mechanism to achieve cell homeostasis [18, 19]. Increasing evidence has revealed that autophagy is involved in cell survival, aging, and homeostasis. In addition, it has been shown that autophagy disorders are involved in various diseases, such as cancer, neurodegeneration, and metabolic diseases [20–22]. Previously, it has been shown that autophagy was associated with the pathogenesis of inflammatory diseases, including OA [23]. In the initial degenerative phase, autophagy in OA chondrocytes is increased to protect chondrocytes from adaptive responses to various environmental changes. Subsequently, as cartilage gradually degenerates, autophagy is reduced, which is associated with cell death [24]. In addition, the PI3K/AKT/mTOR pathway is widely recognized as a fundamental intracellular signaling pathway that takes part in normal cellular physiology and abnormal pathology [25, 26], and can inhibit autophagy when activated [27]. In many studies, the role of inflammatory factors has been shown in immunity, thereby affecting the pathogenesis of inflammatory diseases including OA, and IL-17A was found to be the key inflammatory factor [28]. In several studies, it was demonstrated that IL-17A influenced autophagy in a cell-dependent manner: autophagy was inhibited in epithelial cells in the liver and lungs, while autophagy was induced in RAW macrophages and B cells [29, 30]. Moreover, interactions between IL-17A and the PI3K/AKT/mTOR signaling pathway were investigated in several diseases: the effect on autophagy in psoriasis; induced proliferation and migration of glioma cells; the role of lipopolysaccharide (LPS)-induced acute lung injury, etc. [31–33]. However, studies on clarifying the mechanism of action of ciRS-7/miR-7 axis on the destruction of OA cartilage by autophagy and PI3K/AKT/mTOR signaling pathways mediated by IL-17A have not yet been performed. In this study, we further investigated the role of the ciRS-7/miR-7 axis in OA. Taken together, our findings suggest that the ciRS-7/miR-7 axis provides valuable information for novel treatment strategies in OA.

## RESULTS

### Cartilage degradation and defection of autophagy were validated in OA

**Figure 1:**
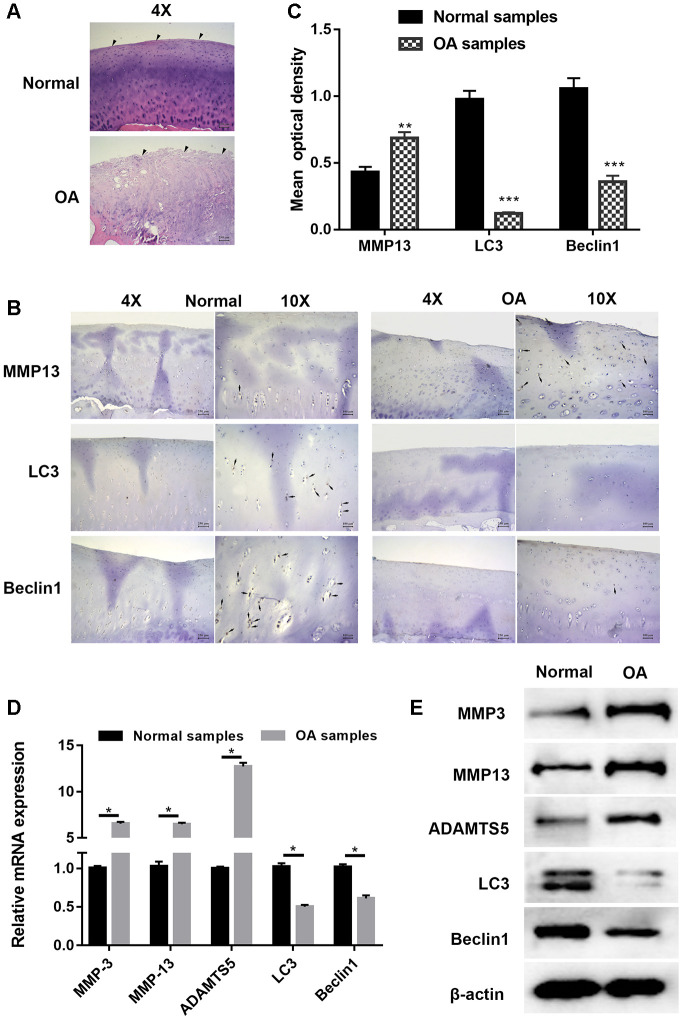
**Cartilage degradation and defection of autophagy were validated in osteoarthritis samples.** (**A**) Representative images hematoxylin and eosin (HE) staining; (**B**) Representative images of immunohistochemistry; (**C**) Quantitative optical density analysis of immunohistochemistry for samples; (**D**) mRNA and (**E**) protein expression of cartilage related proteins (MMP3, MMP13, and ADAMTS5) and autophagy related proteins Beclin1 and LC3. Data represent the mean ± SD (n=6), ** *p* < 0.01 and *** *p* < 0.001 vs. control samples.

Articular cartilage samples of normal and OA patients were employed for histological and immunohistochemical analysis. Hematoxylin and eosin (HE) staining shown in Figure 1A demonstrates that cartilage in OA samples was severely degraded (wearing surface, fissuring in matrix, decreased chondrocytes) when compared with normal tissues in. The immunohistochemistry results displayed in Figures 1B and 1C show that the signal of MMP13, a well-known cartilage degradation-related protein, was remarkably increased, while that of the autophagy-related protein LC3 was significantly reduced. As the substrate of LC3, immunohistochemical staining for Beclin1 showed a similar trend. PCR and Western blot analysis shown in Figure 1D and 1E also revealed that the expression of cartilage degradation-related and autophagy-related proteins was significantly changed. Taken together, these findings indicated that cartilage degradation and autophagy defects were observed in OA samples when compared with normal samples.

### IL-1β exposure induced defection of autophagy in chondrocytes

**Figure 2:**
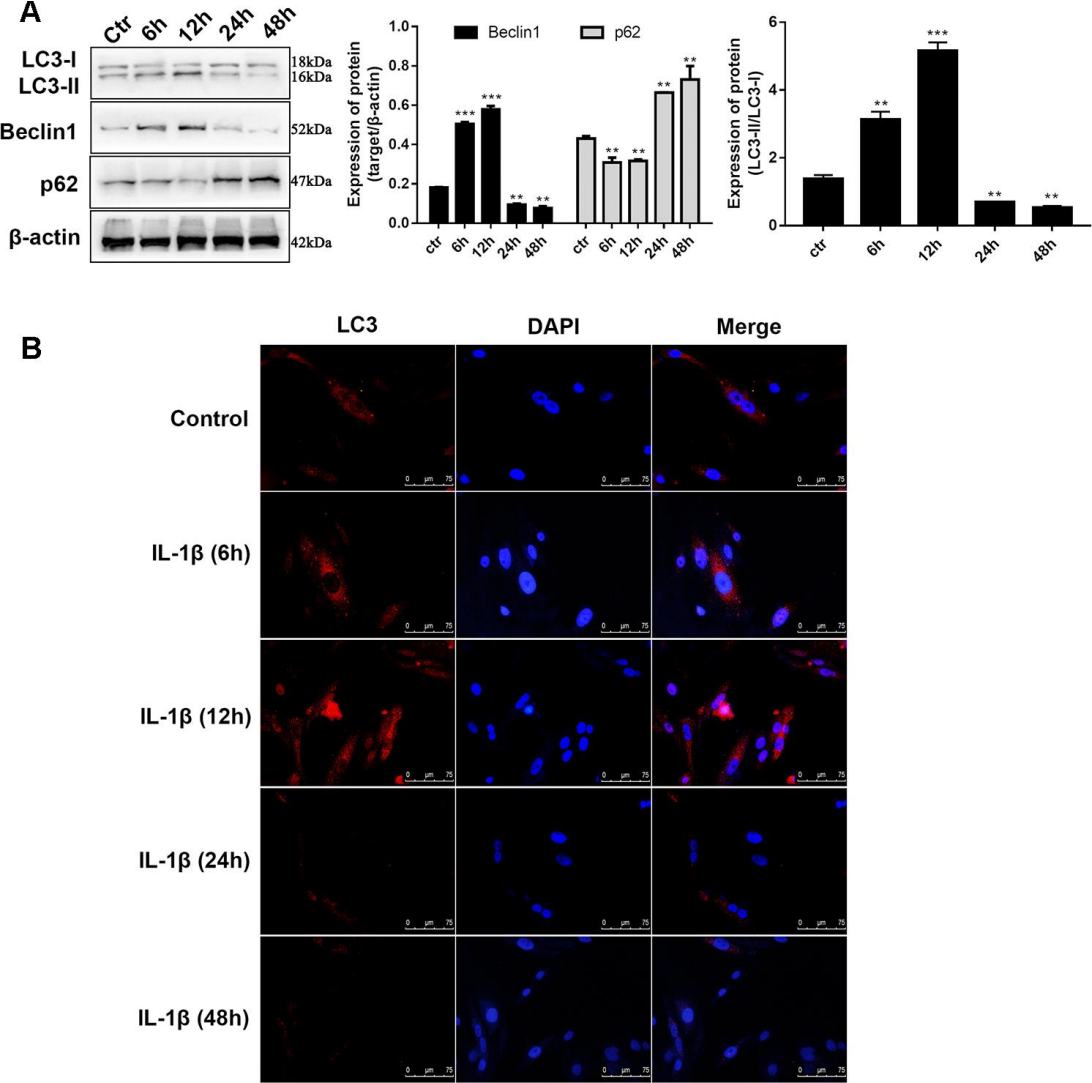
**IL-1β exposure induced defection of autophagy in chondrocytes.** (**A**) Western blot analysis of IL-1β-induced chondrocytes at different time points (6, 12, 24, 36, and 48 h) using antibodies directed to LC3, p62, and Beclin1 and normalized to β-actin. Data represent the mean ± SD (n=5), ** *p* < 0.01 and *** *p* < 0.001 vs. unstimulated cells; (**B**) Representative immunofluorescence photomicrograph of LC3 (red)-labelled chondrocytes. Nuclei were stained with DAPI (blue).

In previous reports, 10 ng/mL of IL-1β was selected to simulate the OA environment in chondrocytes. The indicators of autophagy were determined at different time intervals to reveal whether IL-1β influences autophagy in chondrocytes. Western blot analysis of LC3, an autophagic marker, is the most common method for assessing autophagy. During autophagy, cytosolic LC3 (LC3-I) will be converted to autophagosome membrane type LC3-II. The proportion of LC3-II/I has a prospective effect on the autophagy level. We observed that during the time monitoring process, the LC3-II/I level varied with time. Compared to unstimulated C28/I2 chondrocytes, LC3-II/I levels increased 3.4-fold after 12 hours of stimulation whereas the expression decreased by 3.7-fold after 24 hours and continued to decrease until 48 hours (Figure 2A). Similar to LC3-II, we found that after 12 hours of stimulation, the Beclin1 level increased to 2.7-fold, and after 24 hours the level reduced to about half of that of the control group. The changes in level of the autophagy-related protein p62 was opposite to that of Beclin1 (Figure 2A), which can be explained because p62 is a substrate for LC3 that degrades during autophagosome-lysosomal fusion. Immunofluorescence assay was utilized to determine the intensity of LC3 in IL-1β-stimulated chondrocytes at different time points. Figure 2B shows that the fluorescence intensity of LC3 was significantly enhanced at 6h and 12h, and was significantly lower after 24h when compared with the control group. Taken together, these results validated the findings presented in previous studies that the autophagy behavior of chondrocytes is ultimately inhibited in the OA process. Whether ciRS-7/miR-7 axis affects OA via autophagy demands further research.

### The ciRS-7/miR-7 axis regulates interleukin 1β-stimulated cartilage degradation and defection of autophagy *in vitro*

**Figure 3:**
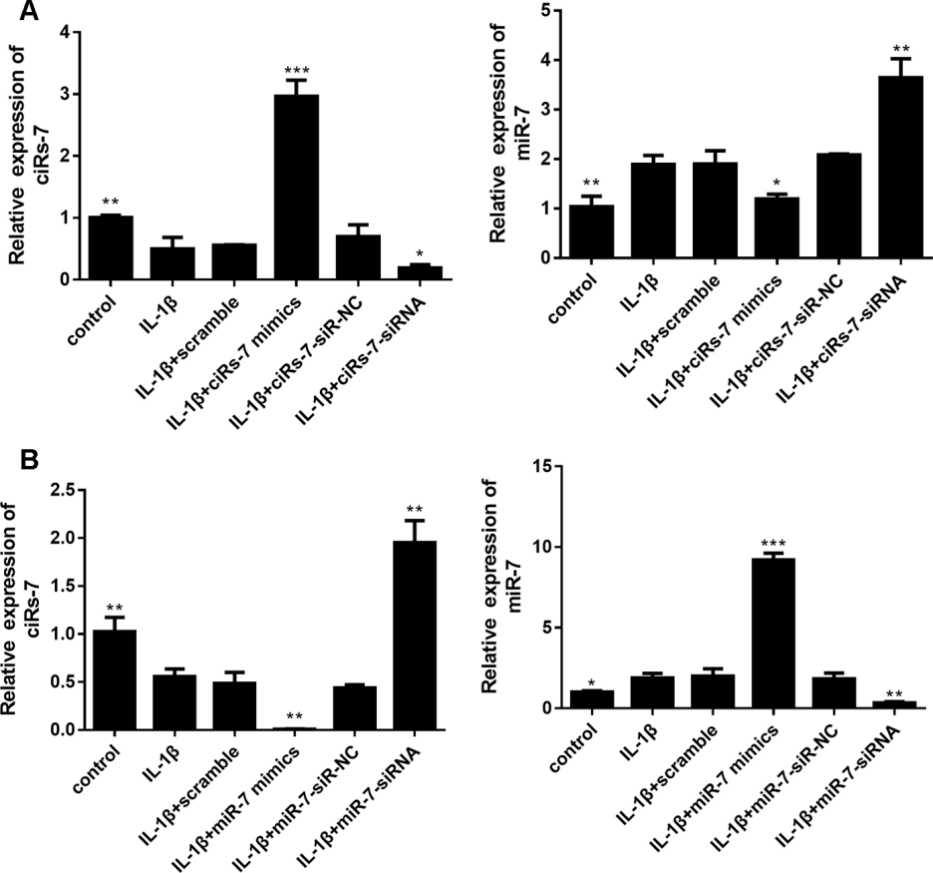
Levels of ciRS-7 and miR-7 in chondrocytes with different transfections: ciRS-7 related transfections (**A**) and miR-7 related transfections (**B**). Data represent the mean ± SD (n=3), * *p* < 0.05, ** *p* < 0.01 and *** *p* < 0.001 vs. the IL-1β group.

In this study, isolated chondrocytes underwent different transfections. As indicated in Figure 3, levels of ciRS-7 and miR-7 were remarkably changed after transfection.

**Figure 4:**
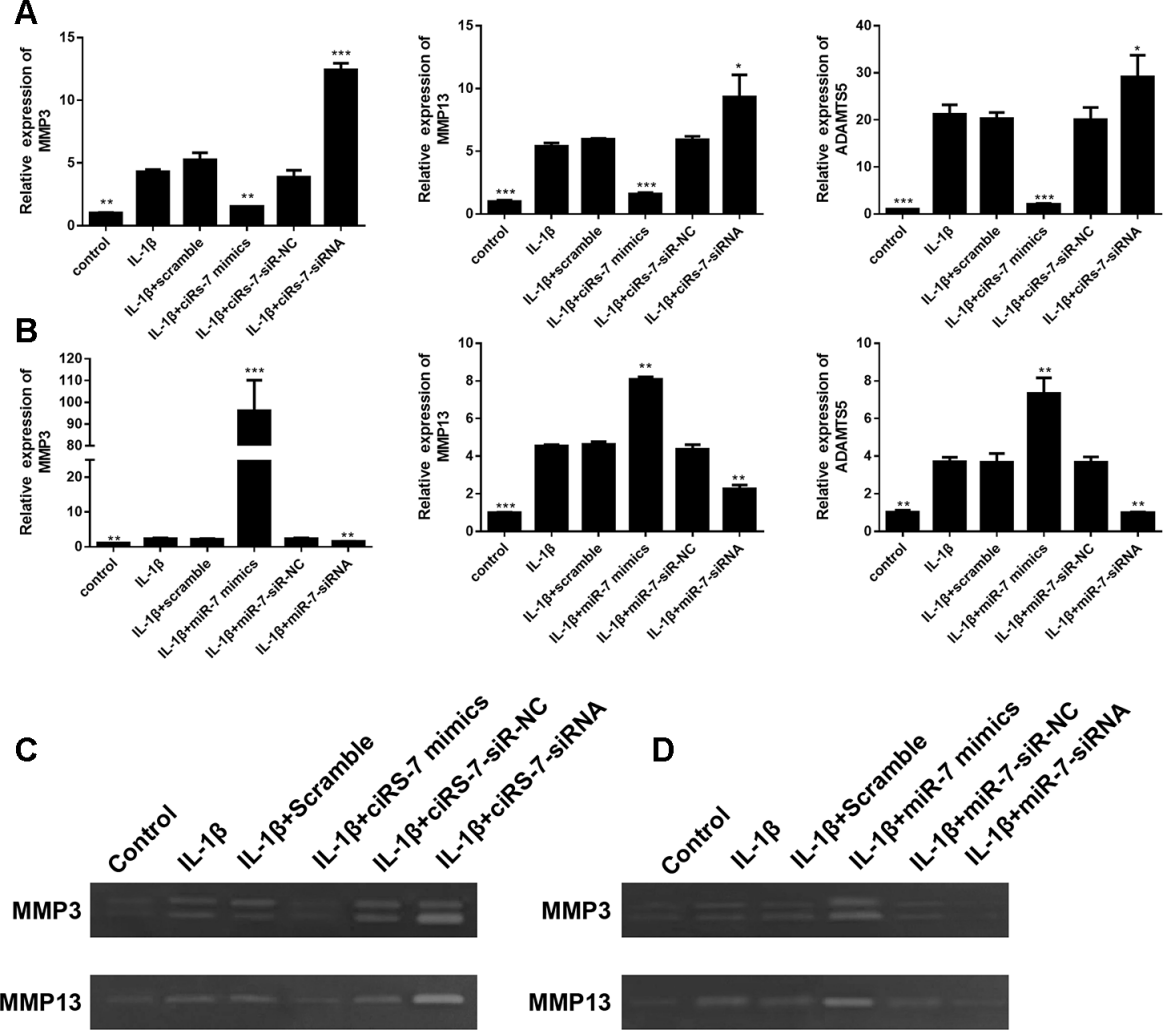
Effects of ciRS-7-related transfections (**A**) and miR-7-related transfections (**B**) on mRNA levels of cartilage-related genes MMP3, MMP13, and ADAMTS5; (**C**) and (**D**) Zymographic analysis of the effects of ciRs-7 or miR-7 on enzymatic activities of MMP3 and ADAMTS5. Data represent the mean ± SD (n=3), * *p* < 0.05, ** *p* < 0.01 and *** *p* < 0.001 vs. the IL-1β group.

**Figure 5:**
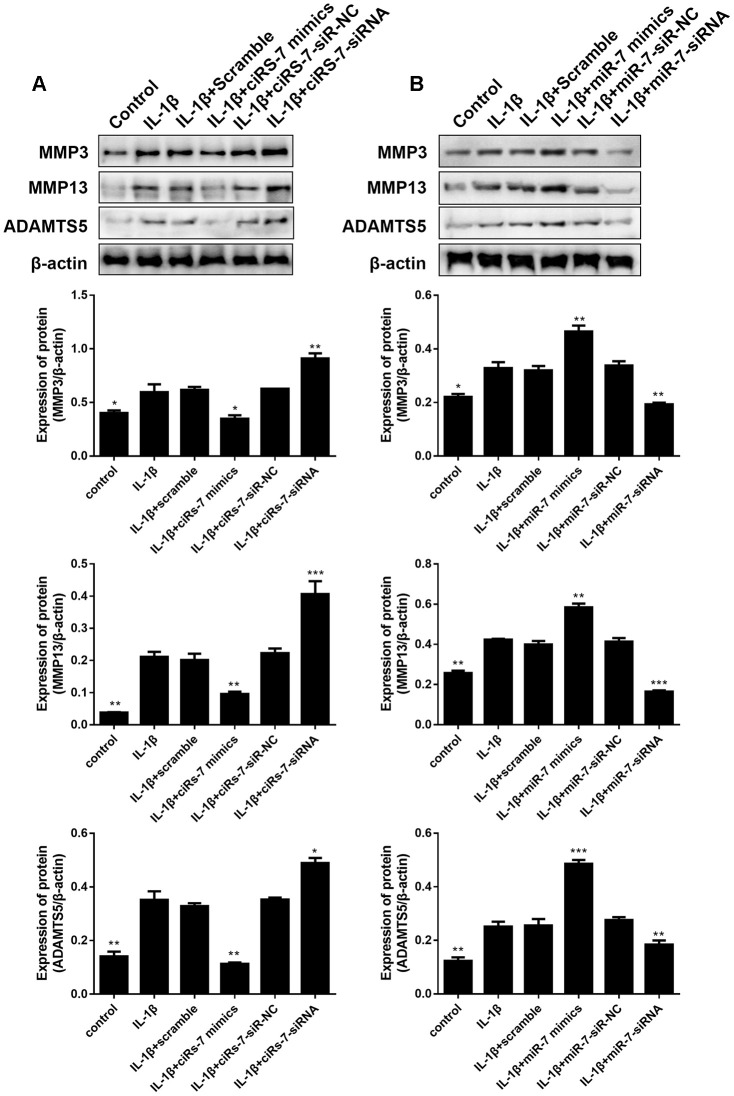
Effects of ciRS-7-related transfections (**A**) and miR-7-related transfections (**B**) on protein levels of cartilage-related MMP3, MMP13, and ADAMTS5. Data represent the mean ± SD (n=3), * *p* < 0.05, ** *p* < 0.01 and *** *p* < 0.001 vs. the IL-1β group.

To determine the mechanism of the ciRS-7/miR-7 axis on IL-1β-stimulated chondrocytes, its effect on cartilage matrix-related MMP3, MMP13, and ADAMTS5 in OA chondrocytes was determined by qRT-PCR and Western blot analysis. The results showed that highly expressed ciRS-7 significantly reduced the expression of MMP3, MMP13, and ADAMTS5 in OA chondrocytes both at the mRNA level (Figure 4A) and the protein level (Figure 5A), while no significant effect was observed on unstimulated cells. Low levels of miR-7 had a similar effect on cartilage matrix-related proteins (Figures 4B and 5B). Gelatin zymography was used to further examine whether the ciRS-7/miR-7 axis could affect the enzymatic activity of MMP3 and MMP13. The gelatinolytic activity of MMP3 and MMP13 was significantly suppressed by highly expressed ciRS-7 and low levels of miR-7 (Figure 4C, 4D). Considering the key role of MMPs and ADAMTS5 in cartilage degradation, the above results indicated that highly expressed ciRS-7 and low levels of miR-7 can reverse the upregulation of MMP3, MMP13, and ADAMTS5 induced by IL-1β stimulation, thereby protecting chondrocytes from inflammatory degradation.

**Figure 6:**
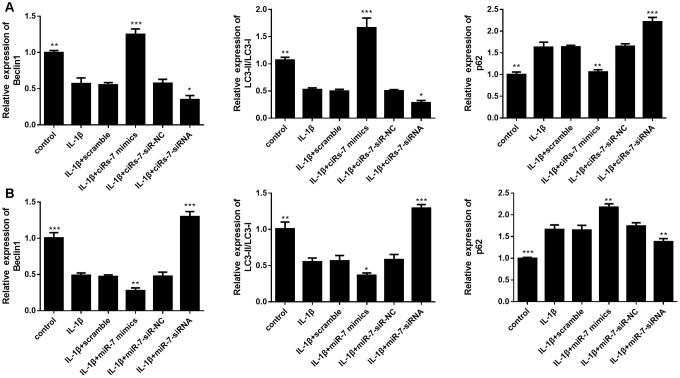
Effects of ciRS-7-related transfections (**A**) and miR-7-related transfections (**B**) on mRNA levels of autophagy-related genes Beclin1, LC3-II/I, and p62. Data represent the mean ± SD (n=3), * *p* < 0.05, ** *p* < 0.01 and *** *p* < 0.001 vs. the IL-1β group.

**Figure 7:**
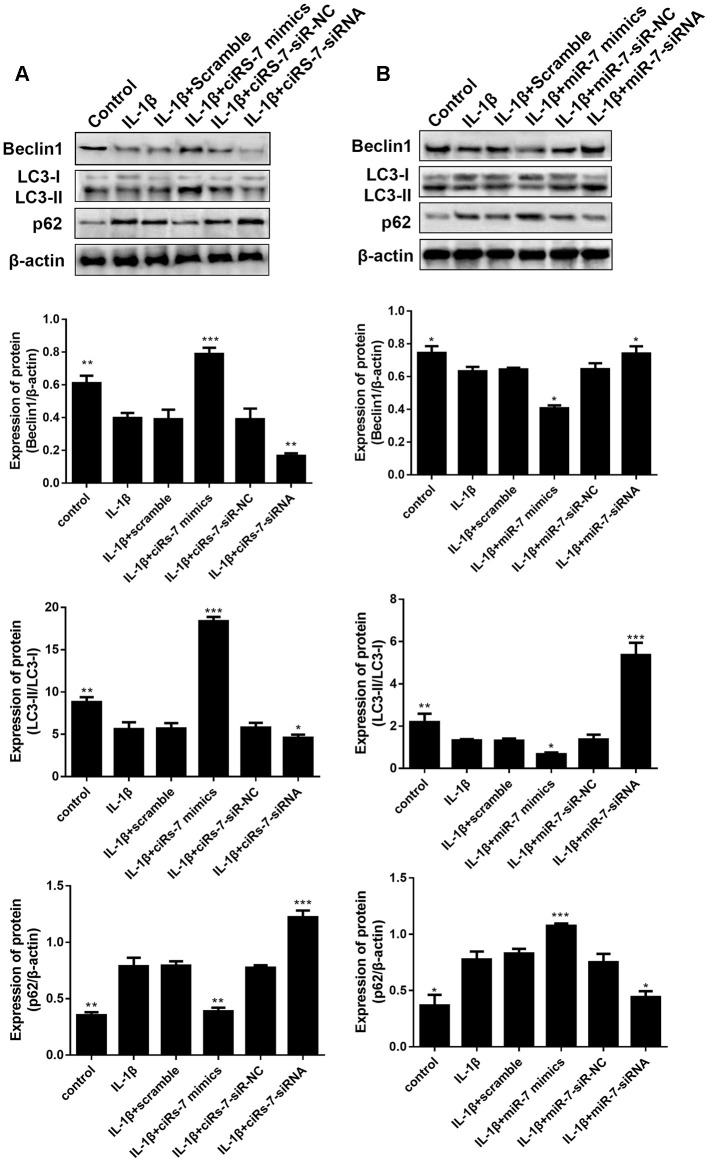
Effects of ciRS-7-related transfections (**A**) and miR-7-related transfections (**B**) on protein levels of autophagy-related Beclin1, LC3-II/I and p62. Data represent the mean ± SD (n=3), * *p* < 0.05, ** *p* < 0.01 and *** *p* < 0.001 vs. the IL-1β group.

Furthermore, we evaluated the function of the ciRS-7/miR-7 axis on autophagy inhibition in OA chondrocytes. We performed qRT-PCR and Western blot analysis to show that highly expressed ciRS-7/low levels of miR-7 significantly increased expression of the autophagy-related gene Beclin1 at both mRNA and protein levels when compared with the IL-1β-induced group, and no changes were observed in unstimulated chondrocytes (Figures 6 and 7). The ciRS-7/miR-7 axis also significantly restored the conversion of LC3-I to LC3-II, which was significantly inhibited by IL-1β (Figures 6 and 7), while p62 had the opposite effect on LC3-II. In conclusion, the above findings confirmed that low levels of ciRS-7/highly expressed miR-7 axis aggravated IL-1β-induced autophagy inhibition in chondrocytes.

### Identification of differentially expressed mRNAs and gene set enrichment analysis.

How the ciRS-7/miR-7 axis influences the autophagy process by regulating related molecules and pathways deserves further studies. Gene sequencing and bioinformatics analysis were performed on three groups of cells, including A: control group, B: IL-1β group, and C: IL-1β+miR-7-mimics group.

**Figure 8:**
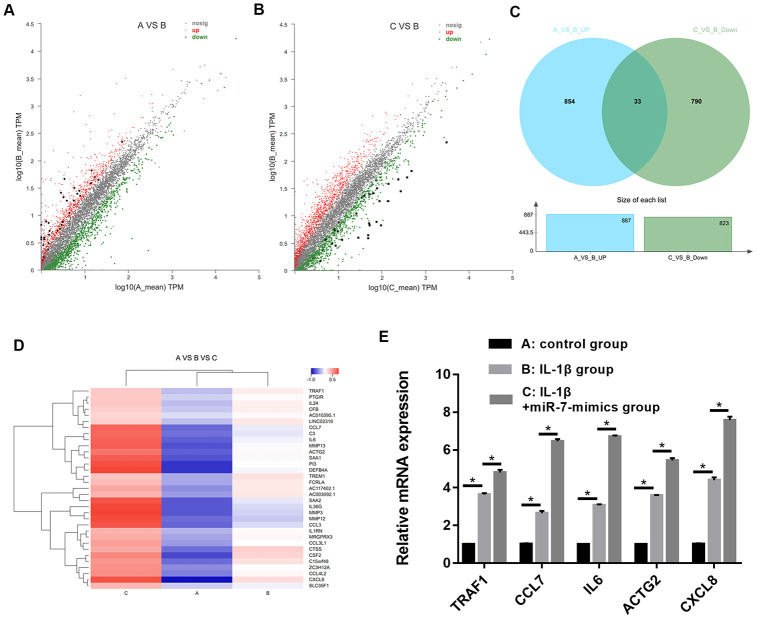
Scatter plot of differential expression of A vs. B (**A**) and C vs. B (**B**). Every point in the plot indicates the expression of a gene in the two experiments, and the red and green points in the plot represent the upregulated and downregulated mRNAs with statistical significance, respectively. Black points represent the 33 overlapping mRNAs between the above-mentioned upregulated genes and downregulated genes. The X-axis and Y-axis represent the log10 TPM values in the two experiments, respectively. (**C**) Venn diagram comparing differentially-expressed genes (DEGs) between A vs B and C vs B. Indicated in the diagram are the numbers of upregulated and downregulated DEGs. (**D**) Cluster analysis of overlapping genes expressed in groups A, B, and C. (**E**) RT-PCR results for five random differentially expressed mRNAs (TRAF1, CCL7, IL6, ACTG2, and CXCL8). Data represent the mean ± SD (n=3), * p < 0.05.

To identify the most significant candidates, mRNAs with at least a 2.0-fold change in expression and p-value ≤ 0.05 were selected. A total of 887 mRNAs (Figure 8A) were up-regulated in group B compared to group A. Simultaneously, the expression of 823 mRNAs (Figure 8B) was down-regulated in group B compared to group C. Moreover, we found that 33 mRNAs were overlapped between the above-mentioned up-regulated and down-regulated genes (Figure 8C). Cluster analysis of the expression of the 33 mRNAs identified 7 distinct groups involved in inflammatory and immune responses (Figure 8D). The random five differentially expressed mRNAs (TRAF1, CCL7, IL6, ACTG2, and CXCL8) were confirmed by RT-PCR (Figure 8E).

**Figure 9:**
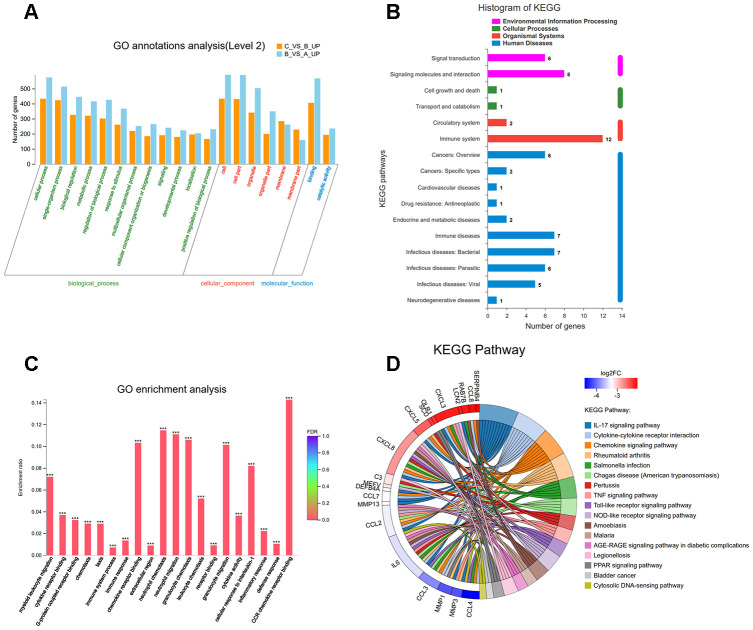
(**A**) Classification of 33 overlapping genes in accordance with gene ontology (GO) categories: biological process, cellular component, and molecular function; The vertical axis represents numbers of differentially-expressed genes (DEGs) corresponding to the numbers of GO terms assigned for a particular GO category. (**B**) KEGG pathway analysis identified that KEGG categories were enriched in 33 overlapping genes. (**C**) GO terms enrichment analysis for 33 overlapping genes. (**D**) Circos plot showing the relationship between KEGG pathway and their genes. Genes are located on the left side of the graph and indicated by their symbols. Gene involvement in the KEGG pathways is indicated by connecting lines.

To understand the function of the up- and down-regulated genes that correspond to C vs B and A vs B groups, genes were mapped to terms in the gene ontology (GO) database to search for significantly enriched GO terms compared to the reference gene background. GO functional enrichment analysis was performed, thereby adjusting p-value of < 0.05 as the cutoff. The differentially expressed genes (DEGs) were identified into 12 categories of “biological process” (Figure 9A). The most abundant categories were “cellular process”, “single-organism, and “biological process”. In the molecular function classification, only “binding” and “catalytic activity” were significantly overrepresented. Under the classification of cellular component, “cell,” “cell part,” and “organelle” were prominently represented. Kyoto Encyclopedia of Genes and Genomes (KEGG) pathway analysis of 33 DEGs revealed that 12 genes were enriched in the “immune system” pathway. Moreover, we identified “signal transduction” and “signaling molecules and interaction”-related pathways were also overrepresented (Figure 9B). GO function enrichment analysis showed that immune response-related terms were also top ranked, such as “CCR chemokine receptor binding”, “neutrophil chemotaxis”, “neutrophil migration”, and “granulocyte chemotaxis” (Figure 9C). In addition, detailed pathway analysis indicated that 7 of the 33 mRNAs were enriched in the IL-17 signaling pathway (DEFB4A, MMP13, MMP3, IL6, CXCL8, CSF2, and CCL7). The Top 3 KEGG pathways were “IL-17 signaling pathway”, “cytokine-cytokine receptor interaction”, and “rheumatoid arthritis”, respectively (Figure 9D).

### Down-regulated ciRS-7/up-regulated miR-7 axis aggravated cartilage degradation and defection of autophagy by PI3K/AKT/mTOR activation mediated by IL-17A in IL-1β-induced chondrocytes

The interaction and functions between ciRS-7 and miR-7 were validated after different transfections. Only miR-7-related transfections (scramble, miR-7 mimics, siR-NC, and miR-7-siRNA) were employed for the following studies.

**Figure 10:**
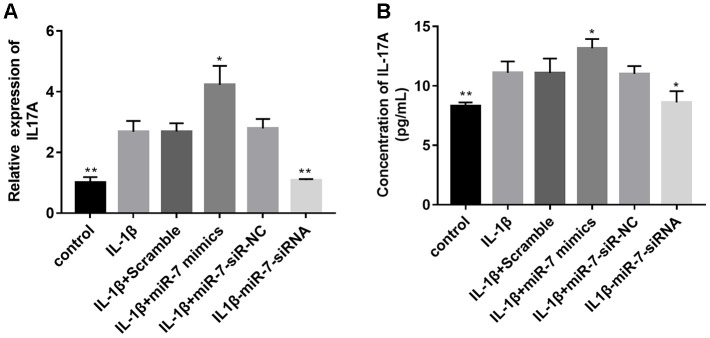
Effects of miR-7 related transfections on IL-17A levels determined by qRT-PCR (**A**) and ELISA (**B**). Data represent the mean ± SD (n = 3), * *p* < 0.05 and ** *p* < 0.01vs. IL-1β group.

Based on the above bioinformation analysis, the IL-17 signaling pathway has changed significantly in the IL-1β group and IL1β+miR-7 mimics group and plays a key role. Within the IL-17 family, IL-17A is the most intensively studied cytokine. Therefore, we first tested the expression of IL-17A in different groups by qRT-PCR and ELISA assay. Figure 10 shows that the expression of IL-17A in the IL-1β+miR-7-mimics group was significantly higher when compared to that in the IL-1β group, and that expression of IL-17A in the IL1β+ miR-7-siRNA group was significantly lower and not significantly different when compared with the control group, thereby indicating that miR-7-siRNA restored IL-17A expression to normal levels.

**Figure 11:**
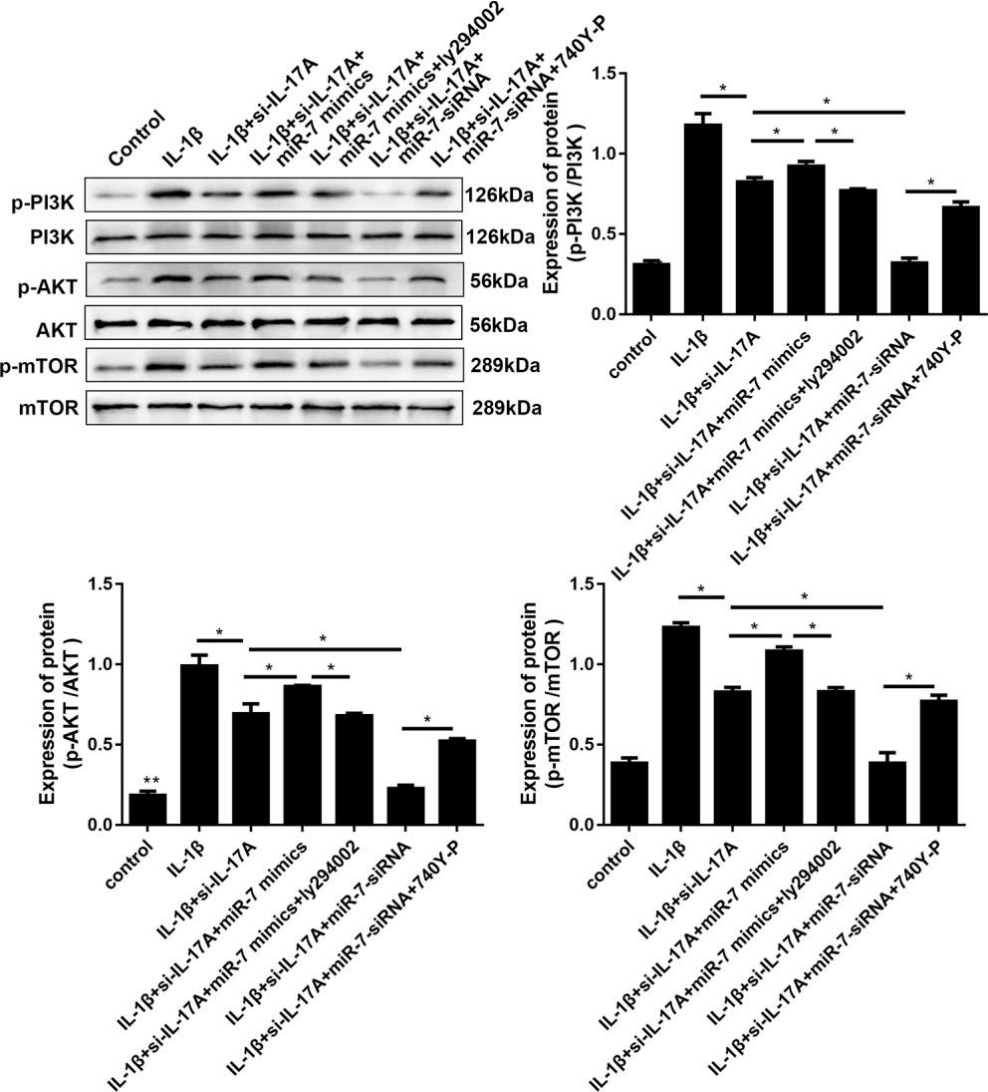
**Effects of miR-7-related transfections mediated by IL-17A on activation of PI3K/Akt/mTOR signaling. Representative Western blot analysis and band intensity analysis of different treatment groups.** Data represent the mean ± SD (n = 3), * *p* < 0.05.

It has previously been reported that elevated IL-17A can activate the PI3K/AKT/mTOR pathway to inhibit autophagy. Therefore, involvement of IL-17A in IL-1β-induced cartilage degradation and defection of autophagy by PI3K/AKT/mTOR activation by miR-7 was further investigated. As shown in Figure 11, IL-1β remarkably increased phosphorylation levels of PI3K, Akt, and mTOR, whereas si-IL-17A treatment significantly inhibited IL-1β-associated phosphorylation of the PI3K/AKT/mTOR pathway. After co-transfection with miR-7 mimics, phosphorylation levels increased, while ly294002 (a PI3K inhibitor, Sigma, St. Louis, MO, USA) reduced this increase. MiR-7-siRNA and 740Y-P (a PI3K activator, Sigma, St. Louis, MO, USA) had opposite effects, all of which suggested the involvement of IL-17A in IL-1β-induced cartilage degradation, and defection of autophagy by miR-7 was related to regulating the phosphorylation of the PI3K/AKT/mTOR pathway.

**Figure 12:**
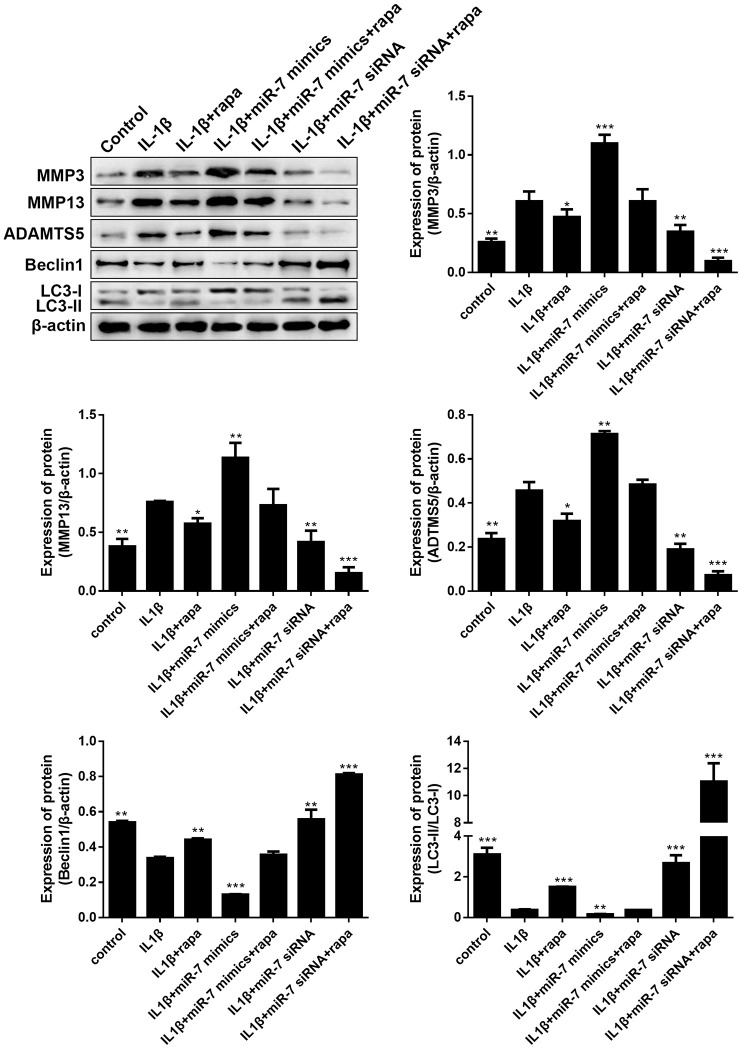
**Effects of the PI3K/Akt/mTOR pathway on the expression of cartilage degradation-related proteins and autophagy-related proteins in IL-1β-induced chondrocytes.** Representative Western blot analysis and band intensity analysis showing protein levels of MMP3, MMP13, ADAMTS5, Beclin1, and LC3 in chondrocytes that received different treatments. Data represent the mean ± SD (n = 3), * *p* < 0.05, ** *p* < 0.01 and *** *p* < 0.001 vs. the IL-1β group.

Given that the PI3K/Akt signaling pathway is another key upstream inhibitor of autophagy, rapamycin (rapa), a potent mTORC1 inhibitor and well-known inducer of autophagy, was studied to further demonstrate the role of the PI3K/AKT/mTOR pathway in OA. As indicated in Figure 12, treatment with rapa further blocked the matrix degradation and autophagy inhibition caused by IL-1β, promoted the conversion of LC3-I to LC3-II in OA chondrocytes, enhanced the expression of autophagy-associated Beclin1 proteins, and significantly reduced the expression of matrix degradation related proteins, including MMP3, MMP13, and ADAMTS5. In addition, rapa can synergistically enhance the protective effect of miR-7-siRNA-treated OA chondrocytes (reduced the expression of matrix degradation-related proteins and restored autophagy activity). These results indicated that down-regulated ciRS-7/up-regulated miR-7 axis weakened the autophagy ability of OA chondrocytes by regulating the PI3K/Akt/mTOR pathway, the functional negative regulator of autophagy (Figure 12).

### Down-regulated ciRS-7/up-regulated miR-7 axis aggravated cartilage degradation and defection of autophagy in rat OA models

**Figure 13:**
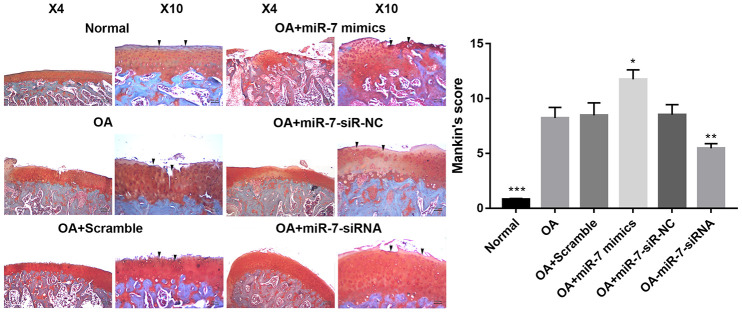
**Representative images of safranin O-stained rat knee joint sections with different treatments and the related Mankin’s scores of each group.** Data represent the mean ± SD (n = 10), * *p* < 0.05, ** *p* < 0.01 and *** *p* < 0.001 vs. IL-1β group.

Given the findings of ciRS-7/miR-7 axis *in vitro*, a rat OA model was developed to evaluate the corresponding effects *in vivo*. Lentiviruses with different encoding genes were injected into the knee joints of OA rats. Cartilage was collected for histological evaluation. Significantly reduced Safranin O staining and severe cartilage destruction was discovered in the OA+miR-7 mimics group when compared with the OA group. Intra-articular injection of miR-7-siRNA-expressing lentiviruses remarkably alleviated surgical resection-induced cartilage destruction (Figure 13). Similar results were observed in the Mankin’s score, suggesting that miR-7-siRNA treatment significantly attenuated OA in rats when compared with the OA group (*p*<0.01).

**Figure 14:**
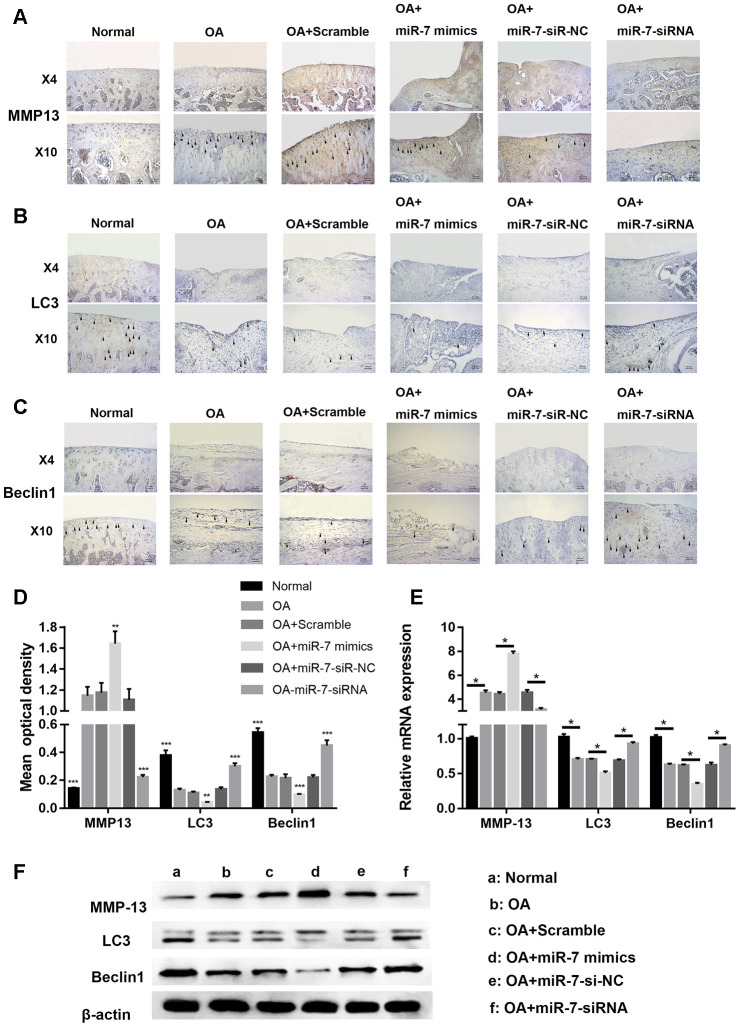
Representative images of immunohistochemical staining for (**A**) MMP13, (**B**) LC3, and (**C**) Bcelin1; (**D**) Quantitative optical density analysis of immunohistochemical staining for different groups; (**E**) mRNA and (**F**) protein expression of MMP13, LC3, and Beclin1. Data represent the mean ± SD (n = 10), ** *p* < 0.01 and *** *p* < 0.001 vs. the IL-1β group.

Immunohistochemical analysis for cartilage degradation- and autophagy-related proteins demonstrated that the relative optical density of the MMP13 protein in the OA+miR-7-siRNA group was significantly lower when compared to that in the OA group (*** *p* < 0.001, Figure 14A and 14D), while that of the OA+miR-7 mimics group was significantly higher when compared to that in the OA group (** *p* < 0.01, Figure 14A and 14D). The relative optical density of LC3 or Beclin1 was significantly increased in the OA+miR-7-siRNA group when compared with the OA group (*** *p* < 0.001, Figure 14B–14D), and was similar to the normal group. The mRNA and protein expression of MMP13, LC3, and Beclin1 showed a trend that was similar to the immunohistochemistry results (Figure 14E and 14F). In accordance with the results, it was concluded that down-regulated ciRS-7/up-regulated miR-7 axis aggravated cartilage degradation and defection of autophagy in the rat model of OA.

## DISCUSSION

OA is a common and complex joint disease that involves inflammation in the cartilage, bone, and synovium [2]. The released inflammatory factors play a key role in OA, and IL-1β is widely recognized as the major catabolic mediator in the pathogenesis of OA [34, 35]. IL-1β is commonly utilized to induce OA models *in vitro*, and was therefore employed in this study to mimic OA pathophysiology.

Autophagy is a highly controlled process, and defects in autophagy have been associated with various diseases, including neurodegeneration, aging, infectious diseases, and inflammatory diseases [36]. As a negative regulator of inflammation, autophagy is thought to be a protective mechanism in normal cartilage, and decreased expression of key autophagy genes has been associated with increased apoptosis during OA development [37]. In this study, we showed that after the addition of IL-1β, the autophagy activity was activated in chondrocytes (6h, 12h), and was significantly inhibited after 24h and continued this autophagy inhibition state. Chondrocytes that were stimulated for 24h with IL-1β were considered successful OA cell models, in which autophagy was significantly inhibited.

In our previous study, we showed that expression of the ciRS-7/miR-7 axis in OA was abnormal, and low levels of ciRS-7/highly expressed miR-7 further aggravated the release of inflammatory factors in C28/I2 chondrocytes, inhibited the proliferation of chondrocytes and promoted apoptosis of chondrocytes [17]. The aim of this study was to investigate the underlying mechanism of action of the ciRS-7/miR-7 axis in OA.

Destruction of extracellular matrix (ECM) homeostasis is a key event in the pathogenesis of OA [38]. Because of their ability to degrade various ECM components, MMP-13 and ADAMTS5 are two key factors in ECM homeostasis [39–41]. In this study, ciRS-7 overexpression/low levels of miR-7 in OA chondrocytes was found to inhibit the expression of MMP3, MMP13, and ADAMTS5, thereby modulating ECM homeostasis leading to OA progression. Low levels of ciRS-7/highly expressed miR-7 further aggravated the generation of MMPs and ADAMTS5 in IL-1β-induced OA cells, which further aggravated degradation of the chondrocyte matrix. ADAMTS5 mediates various functions in tissue remodeling, such as ECM turnover, degradation, catabolism, and destruction, therefore, the ciRS-7/miR-7 axis has broad application prospects in the treatment of OA. Moreover, the role of the ciRS-7/miR-7 axis on autophagy activity was evaluated in several autophagy-related genes both at the mRNA and protein level. The results suggested that highly expressed ciRS-7/low levels of miR-7 can significantly restore impaired autophagy in IL-1β-induced chondrocytes, thereby suggesting a vital function of the ciRS-7/miR-7 axis in the OA cell model: IL-1β-stimulated cartilage degradation can be significantly alleviated by recovering damaged autophagy activity.

We performed RNA-seq for gene expression of three groups of chondrocytes (control group, IL-1β group, and IL-1β+miR-7 mimics group), and found that 33 mRNAs were associated with inflammatory and immune responses, of which 7 were related to the IL-17 signaling pathway, which was verified by KEGG analysis. The IL-17 cytokine family is composed of IL-17A to IL-17F. The most studied family member is IL-17A, which is mainly produced by Th17 cells to protect against extracellular pathogens and fungi, but also has proinflammatory properties. Increased IL-17 has been investigated in synovial fluid and tissues in patients with rheumatoid arthritis [42, 43]. In addition, several animal models have shown that IL-17 has an adverse effect in arthritis [44–46]. Therefore, and based on our RNA-seq analysis, it was concluded that the ciRS-7/miR-7 axis may advance the pathological process of OA by up-regulating IL-17A. In addition, qRT-PCR and ELISA results showed a significant increase in IL-17A expression in OA cells, especially in the miR-7 mimics group. Furthermore, IL-17A expression in the miR-7 inhibitor group was significantly reduced to a level that was almost consistent with that of the control group.

In several studies, it has been demonstrated that IL-17A significantly enriched the mTOR pathway and mediated the proliferation of fibroblast-like synovial cells by mediating this pathway and inflammatory responses in psoriasis [31, 47]. mTOR is centrally regulated by a variety of upstream signaling pathways, including PI3K/Akt signaling, and is a key functional upstream inhibitor of autophagy. The results of this study indicated that IL-17A also affected the PI3K/AKT/mTOR pathway in OA. In OA cells, the expression of p-PI3K, p-Akt, and p-mTOR increased significantly, and the up-regulation of miR-7 exacerbated further activation of this pathway.

Rapa is the most potent and commonly used autophagy inducer, as well as a potent mTORC1 inhibitor [48]. Western blot analysis indicated that after adding rapa to OA cells, part of the autophagy activity was restored and matrix degradation was significantly reduced, possibly by down-regulation of activated mTOR mediated by IL-17A, thereby increasing autophagy. However, how mTOR participates in the regulation of IL-17A signaling in OA requires further investigation.

Based on the *in vitro* results, we tested the hypothesis that intra-articular injection of miR-7 mimics in OA rats maintains ECM homeostasis, and attenuates disease progression *in vivo.* It is evident from the experimental results that the margin of the matrix is smooth and complete, and that autophagy activity is enhanced.

Our study has several limitations. Although this is a classic method for establishing an OA model, it is different from the clinical development process of OA that is caused by many factors. Secondly, in this study, we focused on the effects of the ciRS-7/miR-7 axis on chondrocytes and cartilage, however, we did not study its effects on other cells (fibroblasts, osteoblasts) or tissues (skull, subchondral bone, and meniscus). Therefore, the impact of the ciRS-7/miR-7 axis on OA requires additional in-depth studies.

Taken together, the observations mentioned above suggest that the ciRS-7/miR-7 axis regulates IL-17A-mediated PI3K/AKT/mTOR activation, autophagy damage, and ECM homeostasis in OA, thereby providing novel perspectives and therapeutic strategies for OA.

## MATERIALS AND METHODS

### Human tissue sample collection, HE-staining, and immunohistochemistry

The study protocol was approved by the Ethics Committee of the Affiliated Changzhou No.2 People’s Hospital of Nanjing Medical University (Changzhou, China) and written informed consent was obtained from all participating patients. OA articular cartilage samples and healthy cartilage samples from trauma patients without OA were collected from the Department of Orthopedics of the Affiliated Changzhou No.2 People’s Hospital of Nanjing Medical University (Changzhou, China). For HE-staining, samples were firstly stained with hematoxylin (Beyotime, Nanjing, China) for 5 min. After washing with water, sections were stained with eosin (Beyotime, Nanjing, China) for 5 min, dehydrated with xylene, and mounted in neutral balsam. Next, sections were photographed and observed using a Canon microscopic imaging system (model EOS-350D, Canon, Tokyo, Japan). For immunohistochemistry, hydrated sections were blocked with hydrogen peroxide before pepsin treatment for 20 minutes. Subsequently, sections were blocked with 5% BSA for 1 hour at room temperature and incubated overnight with primary antibodies (Abcam, Cambridge, MA, USA) at 4°C. Then, sections were incubated with horseradish peroxidase-conjugated secondary antibodies (Abcam, Cambridge, MA, USA) for 1 hour at room temperature, and 3,30-diaminobenzidine was used as a chromogenic agent. Immunohistochemical evaluation of OA was performed by three individuals using optical density analysis with ImageJ software (NIH, Bethesda, MD, USA).

### Chondrocytes culture and IL-1β stimulation

C28/I2 chondrocytes were cultured in Dulbecco’s Modified Eagle’s medium (DMEM) (HyClone, USA) containing 1% penicillin/streptomycin (HyClone, USA) and 10% fetal bovine serum (FBS) (Gibco, USA) in an atmosphere with 5% CO_2_ at 37°C. Cells stimulated with IL-1β produced a particularly effective cell model of OA cartilage [49]. A total of 10 ng/ml IL-1β (PeproTech, Rocky Hill, NJ, USA) or PBS was added to C28/I2 cells per well and cells were incubated for an appropriate period. A successful OA model was established when chondrocytes were stimulated with IL-1β for 24 h.

### Immunofluorescence analysis

After reaching over 80% confluence, chondrocytes were shifted to serum-free culture medium for 24 h, and incubated in complete culture medium with 10ng/mL IL-1β for different time periods. Then, PBS was used to wash the cells and cold methanol was added for 20-min to fix the cells. Fixed cells were permeabilized with 0.1% Triton X-100 for 10 min and washed with PBS. Then, a primary antibody directed against LC3 was utilized to treat cells overnight at 4°C in PBS with 1% BSA, then a secondary Alexa Fluor® 594-conjugated antibody (Cell Signaling Technology) was added for 1 hour at room temperature. Cell nuclei were stained with DAPI (Thermo Fisher Scientific) for 5 minutes, then coverslips were mounted on glass slides, and cells were observed with a Leica fluorescence microscope.

### CircRNA and miRNA transfection

A total of 2 × 10^5^ chondrocytes (with or without IL-1β stimulation) was incubated in 2 ml DMEM converged to 80% in a 6-well plate, then serum-free DMEM was added for 12 h. All transfections were conducted based on the manufacturer's instructions. In brief, 100 nM circRNA mimics, miRNA mimics or scramble control (designed and synthesized by Genechem, Shanghai, China) for overexpression experiments, 150 nM ciRS-7 siRNA, miR-7 siRNA, si-IL-17A or negative control siRNA (Genechem, Shanghai, China) for knockdown experiments were mixed with Lipofectamine 2000 (Thermo Fisher Scientific) and left at room temperature for 20 minutes. Before the mixture was added, 1 ml of fresh medium was added to each well, then the mixture was added to the cells for a 24 h transfection.

### Quantitative reverse-transcription PCR (qRT-PCR)

Total RNA was extracted from collected cells using TRIzol Reagent (Invitrogen, Carlsbad, CA, USA), and for real-time qPCR, cDNA was synthesized (Applied Biosystems, Foster City, CA, USA). MiR specific real-time qPCR was conducted following the manufacturer’s guidelines, and U6 small nuclear RNA (snRNA) was used as a control to quantify miRNAs (Clontech, Palo Alto, CA, USA). QRT-PCR was performed using SYBR Green Master Mix (Applied Biosystems, A25780) to determine the expression of mRNAs, using β-actin as an endogenous control. Primer sequences are presented in Table 1.

**Table 1:** The sequences of primers used in this study

**Primers**	**Forward (Sense)**	**Reverse (Antisense)**
**LC3**	GTCACCGGGCGAGTTACC	CTTGAAAGGCCGGTCTGAGG
**Beclin1**	CGAGGTGAAGAGCATCGGG	GCTGTGAGTTCCTGGATGGT
**p62**	ATGAGAGACAAAGCCAAGGAGG	CTCACATGGGGGTCCAAAGA
**miR-7**	TGGAAGACTAGTGATTTTGTT	CCAGTCTCAGGGTCCGAGGTATTC
**ciRs-7**	ACGTCTCCAGTGTGCTGA	CTTGACACAGGTGCCATC
**MMP3**	CTCTTCCTTCAGGCGTGGAT	AGGGAAACCTAGGGTGTGGA
**ADAMTS5**	GCCTCTCCCATGACGATTCC	TCGTGGTAGGTCCAGCAAAC
**MMP13**	CATGAGTTCGGCCACTCCTT	CCTGGACCATAGAGAGACTGGA
**β-actin**	GATGAGATTGGCATGGCTTT	GTCACCTTCACCGTTCCAGT

### Western blot analysis

Western blot analysis was as described in our previous reports [17, 50]. Primary antibodies directed to MMP-3, MMP-13, ADAMTS5, LC3, Beclin1, p62, and β-actin were purchased from Cell Signalling Technology (Danvers, MA, USA) and Abcam (Cambridge, MA, USA).

### Gelatin zymography

Enzymatic activities of MMP3 and MMP13 were assayed by gelatin zymography. Cell lysates of serum-free conditioned medium of different treatments were electrophoresed on 10 % gelatin containing gels (GENMED, Shanghai, China). Subsequently, the gel was washed twice in renaturing buffer (GENMED, Shanghai, China) for 30 minutes, then incubated in developing solution (GENMED, Shanghai, China) for 24h at 37 °C. After staining with Coomassie brilliant blue R-250, enzymatic activities were observed as clear bands.

### Differential gene expression and gene set enrichment analysis

Total RNA was extracted from chondrocytes that underwent different treatments (A: control group, B: IL-1β group, and C: IL-1β+miR-7 mimics group) using Trizol (Invitrogen, Carlsbad, CA, USA) for RNA-seq and was analyzed using Illumina HiSeq platforms. Analysis of differential gene expression was performed with the R package DESeq2. The fold change (≥2.0, corrected p-value ≤ 0.05) was set for up- and down-regulated gene threshold. Differentially expressed genes (DEGs) that showed statistical significance were filtered through a scatter plot. We identified DEGs between untreated cells (group A) and IL-1β treated cell (group B), and DEGs between miR-7 mimics + IL-1β treated (group C) and group B. A Venn diagram showed the overlap between DEGs that were significant in miR-7 overexpressed samples compared with the IL-1β treated group, and samples from the IL-1β-treated group compared with untreated cells. The R package “clusterProfiler” was performed to reveal the functions of the DEGs.

**ELISA assay**

IL-17A levels in culture supernatants of IL-1β-stimulated chondrocytes that underwent different treatments were evaluated using an IL-17A-specific ELISA kit (R&D Systems, Minneapolis, MN, USA) based on the manufacturer’s instructions.

### Lentivirus vector construction

MiR-7 mimics, scramble, miR-7-siRNA, and miR-7-siR-NC cloned into recombinant lentivirus vectors were obtained from Genechem (Shanghai, China). The recombinational and packaging vectors pHelper 1.0 and 2.0 (Genechem, Shanghai, China) were co-transfected into 293T cells with Lipofectamine 2000 to produce viral particles for the following experiments.

### Animal experiments

The destabilized medial meniscus (DMM) model was employed to mimic OA. A total of 30 four-week old male Sprague Dawley rats (200–250 g) of which the medial meniscuses were carefully resected without cartilage and ligament injuries were randomly divided into six groups: Control (no surgery; normal saline treatment, injection time as the OA group; 10 knee joints from 5 rats, n = 10), OA (surgery; normal saline treatment on the first day of every week from the 5^th^ to the 8^th^ week after surgery; 5 rats, n = 10), OA+scramble (surgery; 100 μL normal saline with 1×10^9^ plaque forming units (PFU) of lentivirus vector of scramble treatment, injection time as OA group; 5 rats, n = 10), OA+miR-7 mimics (with surgery;100 μL normal saline with 1×10^9^ PFU lentivirus vector of miR-7 mimics, injection time as the OA group; 5 rats, n = 10), OA+siR-NC (surgery; 100 μL normal saline with 1×10^9^ PFU lentivirus vector of miR-7-siR-NC, injection time as the OA group; 5 rats, n = 10) and OA+miR-7-siRNA (surgery;100 μL normal saline with 1×10^9^ PFU lentivirus vector of miR-7-siRNA, injection time as the OA group; 5 rats, n = 10) treatment groups. After six weeks of treatment, rats were sacrificed by an overdose of anesthesia, and knee samples were obtained and fixed with 4% paraformaldehyde for at least 48 hours. The study was performed according to NIH guidelines (NIH Pub No 85-23, revised 1996), and the protocol was approved by the Ethics Committee of the Second Affiliated Hospital, School of Medicine, Zhejiang University, Hangzhou, China.

### Rat articular cartilage sample preparation, histological analysis, and immunohistochemical analysis

Fixed rat knee joints were decalcified with 10% EDTA-2Na until the samples were soft. Then, gradient dehydration was conducted, followed by embedding in paraffin. Paraffin sections (5 μm) were cut, and subsequently deparaffinized with xylene, hydrated using a gradient, then used for Safranin O-Fast green staining and immunohistochemical analysis. Immunohistochemistry was conducted as mentioned above.

**Statistical analysis**

All quantitative data are presented as the mean ± SD, and were analyzed using SPSS vs. 17.0 (IBM Corporation, Armonk, NY, USA) and GraphPad Prism 7.0 (GraphPad Software, CA, USA). Differences between two groups were analyzed with a t test, while the statistical significance of variances between multiple groups was compared with ANOVA. P < 0.05 was deemed statistically significant.
